# The Significance of Simple Inflammatory Markers in Off Pump Surgery—Review

**DOI:** 10.31083/j.rcm2312400

**Published:** 2022-12-09

**Authors:** Tomasz Urbanowicz, Anna Olasińska-Wiśniewska, Marcin Gładki, Marek Jemielity

**Affiliations:** ^1^Cardiac Surgery and Transplantology Department, Poznan University of Medical Sciences, 61-848 Poznan, Poland; ^2^Pediatric Cardiac Surgery Department, Poznan University of Medical Sciences, 60-572 Poznan, Poland

**Keywords:** OPCAB 1, NLR 2, MLR 3, PLR 4, AISI 5, SII 6, SIRI 7

## Abstract

The inflammatory background of coronary artery disease is gaining more attention in recent times. Off pump surgery is minimally invasive type of surgical revascularization with relatively low number of applications in cardiac surgery centers worldwide that allows for perioperative inflammatory reactions minimalization. The simple inflammatory markers (neutrophil to lymphocyte ratio (NLR), monocyte to lymphocyte ratio (MLR), platelets to lymphocyte ratio (PLR), systemic inflammatory index (SII), systemic inflammatory response index (SIRI), aggregate index of systemic inflammation (AISI)) possess a clinically significant impact on patients’ prognosis and may help to improve patients’ long-term results. The review presents the current knowledge regarding their utility in clinical practice. Assessment of inflammatory indices obtained from whole blood count analysis allows to indicate those patients who need scrupulous follow-up due to predicted worse long-term survival. Perioperative measurement and analysis of simple whole blood counts is inexpensive and easily available and may improve the results of surgical revascularization by better identification of patients at higher risk of worse outcomes.

## 1. Introduction

Coronary artery disease is currently one of the major causes of death related to genetic and environmental factors [1]. Forming healthy lifestyle patterns including physical activity, smoking cessation, proper diet allows for better cardiovascular risk control. Inflammation has a significant contribution of inflammation in atherosclerosis occurrence and progression. The inflammatory background of atherosclerosis due to possible modifiable underdiagnosed characteristics should be regarded as novel approach especially in off-pump coronary artery bypass surgery. A profound biochemical measurement of inflammatory phenomena is difficult and costly, therefore useless in the regular clinical approach. However, the simple inflammatory parameters from whole blood count analysis are easily available and reproducible and may also new perspectives in improvement in patients’ survival.

The aim of current review is to summarize current knowledge on the significance and usefulness of simple inflammatory markers in cardiosurgical revascularization.

The optimal therapy includes pharmacological and interventional approaches. The symptomatic complex coronary artery disease can be treated by either surgical revascularization or percutaneous angioplasty [2, 3]. The surgical revascularization is characterized by favorable long-term outcomes with significant improvement in quality of life [4, 5].

Patients with acute coronary syndromes benefit from well-defined treatment strategies recommended by the current European Society of Cardiology guidelines [6, 7, 8] including surgical revascularization [9]. In chronic stable coronary disease, the results of ISCHEMIA (International Study of Comparative Health Effectiveness with Medical and Invasive Approaches) study brought new perspective on suggested therapeutic approach [10]. In patients with stable coronary artery disease and moderate to severe ischemia in noninvasive stress testing, routine invasive approach failed to reduce major adverse cardiac events (MACE) compared with optimal medical therapy. A benefit regarded those patients with heart failure and left ventricular dysfunction. These findings point out the importance of searching for prognostic markers which would differentiate patients with probable worse prognosis and strong attempts to modify them to achieve better outcomes.

## 2. Advances in Cardiac Surgery

The surgical therapy can be performed as off-pump coronary artery bypass surgery [11] that diminish the risk for inflammatory activations and secondary complications related to cardiopulmonary bypass application [12]. The off-pump surgery is postulated to be related to lower complication risk in perioperative period [13]. The lower atrial fibrillation risk, blood products transfusion and shorter length of hospitalization was documented by Velioglu *et al*. [14]. Elderly population of patients may benefit from this technique in experienced surgical teams [15]. The comparable mid-term major adverse cardiac and cerebrovascular events (MACCE) (HR: 1.03; 95% confidence interval (CI) 0.87–1.24; *p* = 0.714) and survival rates (hazard ratio (HR): 0.91; 95% CI 0.73–1.14; *p* = 0.578) in off-pump and on-pump surgical revascularization were presented insignificant in Sheikhy *et al*. [16]. The comparison of 6574 patients operated with on-pump and 1589 with off-pump techniques revealed comparable results. The primary endpoints which included the 30-days mortality (OR: 1.51; 95% CI 0.93–2.45; *p* = 0.092 in on-pump vs OR 2.02; 95% CI 0.94–4.35; *p* = 0.073 in off-pump, respectively) and 3-years mortality (HR: 1.03; 95% CI 0.87–1.24; *p* = 0.714) after covariate adjustment were not statistically significant though in favor for on-pump technique. The mechanical ventilation time (*p* = 0.003), intensive care unit stay (*p *< 0.001) and overall hospitalization time (*p *< 0.001) were significantly longer in presented analysis in on-pump group. Results of Espinoza *et al*. [17] observational study revealed comparable long-term survival in both techniques in 10 years follow up (off-pump vs. on-pump: 77.9% ± 1.2% vs. 80.2% ± 1.3%, *p* log rank = 0.361). Surgical arterial revascularization despite excellent long-term results [18, 19] accounts currently for only 10% of all surgical revascularization procedures [20]. Multicenter population-based cohort study by Roha *et al*. [21] indicated improved long-term survival and freedom from MACCE. Ongoing ROMA (Randomized Comparison Of The Clinical Outcome Of Single Vs Multiple Arterial Grafts) trial as a multicenter prospective study focused on off-pump arterial revascularization is expected to bring new light on possible optimal surgical therapy [22].

## 3. Off Pump Surgery

Off-pump coronary artery bypass grafting (OPCAB) became one of the most revolutionary technique changes in cardiac surgery. The cardiopulmonary bypass avoidance, although technically challenging, may represent the dominant technique in experienced centers with satisfactory long-term results [23]. About 20% of surgical revascularization procedures worldwide are performed in OPCAB technique [24]. The technique can be regarded as superior for high-risk patients [25] especially in advanced age [26], neurological burden [27, 28], diabetic patients [29, 30] and co-existing chronic obstructive pulmonary disease [31]. It is recommended when end stage kidney failure [32], liver dysfunction [33] or oncologic diseases are co-diagnosed [34, 35].

The off-pump surgery advantages are related to diminish inflammatory activation during the procedure [36]. The perioperative inflammatory activation is postulated to possess detrimental effect related to vasoplegic syndrome [37], mortality [38] and morbidity [39], including stroke risk [40], kidney failure [41] and liver dysfunction [42]. Crucial role of systemic inflammatory limitation in early perioperative morbidity is postulated [43] once the off-pump surgery is performed. It seems to be of outmost importance in aging population. Kuwahara *et al*. [44] in their review suggested that in many observational studies OPCAB long-term results were superior to on-pump surgery excluding acute-phase surgery. Patel *et al*. [45] pointed out the significance of surgeons’ experience and patients’ identification for off-pump technique. Khan *et al*. [46] in their review presented lower in-hospital mortality risk in octogenarians group treated with off-pump technique (pooled OR: 0.64, 95% CI: 0.44–0.93; *p* = 0.02). In randomized EXCEL (Evaluation of XIENCE versus Coronary Artery Bypass Surgery for Effectiveness of Left Main Revascularization) trial, the OPCAB group despite satisfactory perioperative results, was characterized by increased risk of 3-year all-cause death (8.8% vs. 4.5%, HR: 1.94, 95% CI: 1.10–3.41; *p* = 0.02) [47]. The over 15 years of on-pump and off-pump results presented by Deo et al. revealed increased risk-adjusted all-cause mortality (HR: 1.15, 95% CI: 1.13–1.18, *p *< 0.01) [48]. The results of observative study performed by Thakur *et al*. [49] showed increased requirement for repeated revascularization in off-pump patients with 12-month follow-up (OR: 1.59, 95% CI: 1.09–2.33, *p* = 0.02).

## 4. Inflammatory Background

The inflammatory background of atherosclerosis has gained significant attention in recent years [50]. The innate and adaptive immunological mechanism initiating and accelerating inflammatory reactions are believed to trigger atherosclerotic plaques formations and progression [51]. The intramural accumulation of lipoproteins modified by phagocytosis is infiltrated by leukocytes and accompanied by chronic inflammatory response, resulting in plaques formation [52]. Further progression results from release on pro-inflammatory cytokines ad chemokines, enlargement of lipid core and thinning of the fibrous cap [53]. This component of atherosclerosis is the result of individual imbalance in inflammatory hemostasis [54].

Keeping in mind the inflammatory background of coronary artery disease, several clinical studies have been proposed to influence the inflammatory pathways. The Canakinumab Anti-inflammatory Thrombosis Outcomes Study (CANTOS) demonstrated reduction of cardiovascular events in patients with atherosclerosis with the use of neutralizing interleukin-1 beta (Il-1beta) [55]. In the aforementioned study, the relation between neutrophil to lymphocyte ratio (NLR) and clinical outcomes was observed and related to quartiles. The differences between NLR <1.8 and vs NLR >3.09 resulted in increased hazard of MACE by 22% (95% CI: 16–28%, *p *< 0.0001), and CV death by 36% (95% CI: 27–46%, *p *< 0.0001) [56].

Therapeutic targeting of inflammation is much promising. The clinical assessment and surveillance of inflammatory milieu is of priority significance, as well.

Peripheral blood derangements in inflammatory cells counts are now eagerly applied in clinical practice due to their easy accessibility. There is growing evidence presenting the role of simple whole blood counts inflammatory markers in morbidity and mortality prediction in ischemic coronary artery disease [57, 58, 59]. Arbel *et al*. [57] presented the relation between NLR >3.0 and severity of coronary artery disease which was linked to increased risk of worse prognosis (OR: 2.45, 95% CI: 1.76–3.42, *p *< 0.001) and MACE within 3 years following angioplasty (HR: 1.55, 95% CI: 1.09–2.2, *p* = 0.01). The risk of contrast-induced nephropathy following angioplasty in acute coronary syndrome was presented in Butt *et al*. [58] analysis (OR: 2.03, 95% CI: 1.403–3.176, *p *< 0.001; AUC: 0.71).

The whole blood counts analysis revealed simple markers as NLR [56, 60], lymphocyte to monocyte ratio (LMR) [61, 62, 63] and platelets to lymphocyte ratio (PLR) [64] as predictive markers for mortality and major adverse coronary events (MACE) in chronic and acute coronary syndromes. The NLR values measured prior to angioplasty procedures above 3.24 were reported as independent predictor of long-term MACCE (OR: 1.087, 95% CI: 1.026–1.151; *p* = 0.004) in Gurbuz *et al*. [60] analysis. LMR <1.84 in Cai *et al*. [62] study was postulated as significant for MACE prediction in acute coronary syndromes (adjusted hazard ratio [HR]: 1.74, 95% CI: 1.12–2.70; *p* = 0.013). PLR >124–167 was found by Dong *et al*. [63] in their meta-analysis (RR: 2.14, 95% CI: 1.52–3.01, *p *< 0.001, $I^{2}$ = 24%) as all-cause mortality predictor in ST elevation myocardial infarction (STEMI) patients.

The inflammatory indexes based on hematological indices were recently presented as composition of inflammatory cells concentration. The inflammatory response as systemic inflammatory response index (SIRI) combined of neutrophils, monocytes and lymphocytes counts were measured and its prognostic values in coronary artery disease were reported in recent studies. Systemic immune inflammatory index (SII) based on hematological indices (neutrophils and lymphocytes) combined with peripheral thrombocytes counts was shown as a reliable predictor for future events in certain groups of patients with atherosclerotic coronary disease [64, 65] including its severity [66]. SII above 694 × ${\mathrm{10}}^{9}$/L (HR: 1.65; 95% CI: 1.36–2.01, *p *< 0.001; AUC: 0.59) was found as MACE predictor in chronic coronary syndrome [64]. The aggregate index of systemic inflammation (AISI) as a composition of peripheral counts of neutrophils × monocytes × platelets/lymphocytes is a predictor of prolonged stay in thoracic surgery [67]. In the latter analysis of Paliogiannis *et al*. [68] the AISI values above 221 (HR: 1.65; 95% CI: 1.36–2.01, *p* = 0.046; AUC: 0.653) were found significant independent predictor for prolonged hospital stay.

## 5. Inflammatory Activation Prior to Surgery

The inflammatory status prior to surgery is patient dependent [69]. Simple markers obtained from the whole blood count analysis allow for differentiation of patients’ selection with worse long-term prognosis neither related to the completeness of the revascularization nor co-morbidities but associated with individual characteristics. This novel approach based on preoperative hematological indices may enable us to explore why some of patients require repeated interventions or represent a worse prognosis group during follow-up despite successful surgical therapy as presented in Table 1 (Ref. [60, 68, 69, 70, 71, 72, 73, 74]).

**Table 1.** **Preoperative inflammatory indexes and their predictivity for complications following coronary artery bypass grafting**.

Surgical revascularization	Hematological marker value	Predictive role	Statistical significance	References
Preoperative	NLR >4.32	8 years MACE risk in CABG	OR: 1.087, 95% CI: 1.026–1.151, *p* = 0.004; AUC: 0.74	[60]
	NLR >2.51	risk for postoperative AKI in CABG	OR: 1.37, 95% CI: 0.09–1.72, *p* = 0.007; AUC: 0.672	[69]
	NLR >3.46	AF following CABG	OR: 2.62, 95% CI: 1.30–5.29, *p* = 0.001	[70]
	NLR	positive moderate correlation in CABG		[71]
		Hospitalization	r = 0.227, *p* = 0.014	
		ICU stay time	r = 0.220, *p* = 0.014	
	MLR >0.2	5.3 mortality risk in OPCAB	HR: 2.46, 95% CI: 1.33–4.55, *p* = 0.004; AUC: 0.577	[68]
	LMR <2.65	Venous graft thrombosis in CABG	OR: 0.896, 95% CI: 0.465–0.957, *p *< 0.001; AUC: 0.846	[72]
	SII	risk for AF in CABG	OR: 1.002, 95% CI: 1.001–1.002, *p *< 0.01; AUC: 0.711	[73]
	SIRI >1.27	8 years mortality risk in OPCAB	HR: 6.16, 95% CI 2.17–17.48, *p* = 0.012; AUC: 0.682	[74]

Abbreviations: AISI, aggregate index of systemic inflammation; CABG, on pump coronary artery bypass grafting; LMR, lymphocyte to monocyte ratio; NLR, neutrophil to lymphocyte ratio; MLR, monocyte to lymphocyte ratio; OPCAB, off pump coronary artery bypass grafting; PLR, platelets to lymphocyte ratio; SII, systemic inflammatory index; SIRI, systemic inflammatory response index.

The link between preoperative mean values of platelets volume and venous grafts failure [75] indicate the significance of hematologic indices on underdiagnosed hypercoagulability that may emerge an important issue in future approach in cardiac surgery. Currently, the off-pump arterial surgical revascularization represents the low-risk highly effective procedure [76] and focusing on long-term result optimalization should be regarded as the main target for cardiologist/cardiac surgeons.

Preoperative peripheral thrombocytes count and endothelial dysfunction has particular significance, especially in certain groups such as diabetic patients [77]. Simple whole blood count indices may be regarded as poor long-term prognosis indicators that should be considered when patients are referred for surgery to differentiate those requiring special attention or at discharge to plan potentially more aggressive treatment and frequent controls.

## 6. Inflammatory Activation Technique-Related in Perioperative Period

On-pump surgical revascularization possess potential side effect related to inflammatory activation resulting in fluid overload and augmented fluid extravasation [78]. The increased endothelial permeability may be related to non-pulsatile blood flow [79], hemodilution, endothelial activation [80] and hypothermia [81]. Neutrophil related inflammatory response may cause endothelial cells activation [82]. Endothelial cytotoxicity mediated by neutrophils activates intracellular mechanisms of nitric oxide (NO) production [83]. Cardiopulmonary bypass application induces acute inflammatory reaction that recruits neutrophils to the site of tissue injury by complex mediator cascade including tumor necrosis factor (TNF) alfa and IL-1 [84, 85]. Endothelial cells and neighboring parenchymal cells are stimulated by TNF alfa and IL-1 to produce neutrophil-attracting chemokines [32]. Neutrophil adherence and transmigration engaged by adhesion molecules result in transmigration into the extravascular space [86]. During cross-clamping time, the blood supply into the heart is almost completely ceased which induces oxygen radicals’ production (ROS). Moreover, tissue injury is augmented secondary to neutrophils’ activation during reperfusion [87]. Platelets and neutrophils may play a role in so- called “no-reflow phenomenon” [88].

Neutrophils are activated not only when CPB is applied as blood contact with the foreign surface triggers factor XII activation into factors XIIa and XIIf [89]. The contact of the foreign body circuit with the blood components induces inflammatory reactions. The XIIf is responsible for complement complex activation and cell membrane attack, lysis, and cellular death. Moreover, the thrombin, extensively formed during CPB, activates platelets through their thrombin receptors of the PAR family [90]. Activated platelets in turn release neutrophils activators such as Il-6 and Il-8 [91].

OPCAB technique allows to limit inflammatory activation [92] which occurs in 70–90% patients undergoing conventional coronary artery bypass grafting with cardiopulmonary bypass (on-pump) [93]. Systemic inflammatory reactions secondary to on-pump procedures represent the significant risk factor for postoperative multiple organ dysfunction (MOD) [94].

Contemporary achievements in cardiac surgery, including introduction of arterial revascularization and off-pump technique, enable obtaining satisfactory results even in elderly patients, and may be enhanced with prognostic factors identification to improve long term outcomes.

## 7. Blood Samples Timing

In the review we took into consideration either preoperative components of whole blood analysis obtained at the hospital admission or postoperative ones which were mainly focused on the 1st postoperative day. Postoperative analysis is believed to represent reperfusion injury which occurs after revascularization and is related to oxidative stress [95]. The postoperative inflammatory activation related to reperfusion injury following the revascularization procedure is postulated as a prognostic factor in cardiac surgery. The postoperative hematological indices and their prognostic significance depending on off-pump (OPCAB) and on-pump (CABG) technique was presented in Table 2 (Ref. [59, 65, 96, 97, 98, 99, 100]).

**Table 2.** **Postoperative inflammatory indexes and their predictivity for complications following coronary artery bypass grafting**.

Surgical revascularization	Hematological marker value	Predictive role	Statistical significance	References
Postoperative	NLR >4.6	3 years mortality risk in OPCAB	HR: 1.47, 95% CI: 1.3–1.65, *p* < 0.0001, AUC: 0.715	[59]
	NLR >3.5	4.7 years mortality risk in OPCAB	HR: 2.21, 95% CI: 1.48–3.32, *p <* 0.001; AUC: 0.628	[98]
	NLR >8.6	Risk for pericardial effusion	OR: 3.3; 95% CI: 1.56–7.01, *p* = 0.002; AUC: 0.610	[96]
	MLR >1.44	5 years mortality risk in OPCAB	HR: 3.81, 95% CI: 1.45–10.06, *p* = 0.07; AUC: 0.659	[97]
	SII >935	Saphenous graft disease predictor within 1 year following CABG surgery	OR: 3.27, 95% CI 1.94–5.65, *p* < 0.001	[100]
	SII >952	3.7 years mortality risk in DM pts after OPCAB	HR: 3.44, 95% CI: 1.02–11.66, *p* = 0.047; AUC: 0.698	[65]
	SII >878	Poor outcomes risk factor after OPCAB	OR: 1.010, 95% CI: 1.003–1.016, *p* = 0.003; AUC: 0.984	[99]
	SIRI >5.4	4.7 years mortality risk in OPCAB	HR: 0.29, 95% CI: 0.09–0.92, *p* = 0.036; AUC: 616	[98]

Abbreviations: AISI, aggregate index of systemic inflammation; CABG, on pump coronary artery bypass grafting; LMR, lymphocyte to monocyte ratio; NLR, neutrophil to lymphocyte ratio; MLR, monocyte to lymphocyte ratio; OPCAB, off pump coronary artery bypass grafting; PLR, platelets to lymphocyte ratio; SII, systemic inflammatory index; SIRI, systemic inflammatory response index.

## 8. Neutrophil to Lymphocyte Ratio (NLR)

Activated neutrophils release genetic material named neutrophil extracellular traps (NETs) which were found in atherosclerotic plagues [101]. NETs not only induce oxidative stress and endothelial cell dysfunction but promote prothrombotic molecules accumulation [102]. These highly sophisticated markers are difficult to apply in clinical practice but the raise of neutrophil count in peripheral blood may be regarded as simplified method for monitoring of neutrophils activation. Although neutrophils activation can be measured by released cytokines, simple measurement of neutrophil counts derangements in peripheral blood count analysis was proposed as a sufficient marker of inflammation [103, 104, 105], easy to obtain and repetitive.

Neutrophil to lymphocyte ratio (NLR) was proposed as a simple inflammatory marker that possess its value for long-term prognosis in coronary artery disease. Interestingly, it can be preoperatively modified by anti-inflammatory diet [106]. The significance of NLR as prognostic factor following surgical revascularization irrespectively to applied technique (on-pump vs off-pump) was postulated by Şahin *et al*. [107] indicating NLR above 31.8 (*p *< 0.001) in the 1st postoperative day. Publications revealed the impact of inflammatory activation on morbidity risk following coronary artery surgical revascularization. Meta-analysis performed by Shao *et al*. [70] suggested the relation between either preoperative NLR (combined odd ratio for baseline NLR was 1.25 (95% CI: 1.16–1.35, *p *< 0.01) and postoperative NLR (combined odd ratio for baseline NLR was 1.518 (95% CI: 1.076–2.142, *p* = 0.017) and atrial fibrillation. Parlar *et al*. [69] in their analysis corelated the risk of acute kidney failure to NLR above 4 (OR: 1.17, 95% CI: 1.11–1.23; *p *< 0.001). The relation between pericardial effusion and postoperative NLR over 8.6 (OR: 3.3; 95% CI: 1.56–7.01, *p* = 0.002; AUC: 0.610) was presented by Sevuk *et al*. [96]. The postoperative NLR on the 1st postoperative day above 4.6 (HR: 1.47, 95% CI: 1.3–1.65, *p *< 0.0001; AUC: 0.715) was presented as all-cause mortality risk factor within 3 years following OPCAB procedure in 224 pts [59]. The postoperative NLR values above 3.5 were found as marker of poor prognosis in OPCAB group composed of 538 patients and followed for 4.7 ± 1.7 years [98].

The combination of NLR with clinical factors and echocardiographic characteristics was incorporated into predictive score called “OPCAB Predictive Score” [108]. The postoperative NLR increase, together with preoperative clinical factors, enabled identification of high-risk long-term mortality group which should require more scrupulous surveillance.

## 9. Monocyte to Lymphocyte Ratio (MLR)

Blood monocytes play an important role in atherosclerotic plaques formation. The interplay between monocytes and endothelial cells results in local imbalance between two processes (damage and repair) that possess detrimental consequences for plaque development and stability [109]. The primary immune cells present in atherosclerotic plaques are lesion-associated macrophages which form foam cells after cholesterol and lipids accumulation [110]. The monocyte to lymphocyte ratio (MLR) was postulated as predictive factor for saphenous veins patency following surgical revascularization [111]. Inflammatory activation following OPCAB (off-pump coronary artery bypass) measured by MLR >1.44 (HR: 3.81, 95% CI: 1.45–10.06, *p* = 0.07; AUC: 0.659) was proposed as a marker of worse 5-year prognosis [97].

## 10. Platelet to Lymphocyte Ratio (PLR)

Platelets play an important role in acute coronary syndromes by local thrombosis [112] and moreover are involved in atherosclerosis initiation and may sustain vascular inflammation [113]. Thrombocytes help to recruit immune cells such as monocytes and neutrophils in atherosclerosis formation and they appear to act as tissue hemostasis mediators that may modulate the microenvironment of the atherosclerotic plaque [114]. Platelet to lymphocyte ratio (PLR) has been studied as a prognostic factor for cardiovascular diseases. The role of PLR as quickly available and inexpensive marker for a high risk cardiovascular periprocedural adverse events was reported by Serra *et al*. [115] literature review. In Kurtul *et al*. [116] review, the significant role of PLR in acute coronary syndrome presenting the interplay between inflammation, thrombosis and age was presented [116, 117]. Qiu *et al*. [118] in their meta-analysis of 14 studies including 4871 patients, they demostrated relation between higher values of PLR and severity of atherosclerosis in chronic coronary syndromes. Although initially the PLR was reported as perioperative atrial fibrillation risk factor [119], the recent analysis did not support the aforementioned relation [120].

## 11. Systemic Inflammatory Response Index (SIRI)

SIRI combines three inflammatory biomarkers - neutrophils, monocytes, and lymphocytes in the peripheral blood count. Its advantages are related to comprehensive characteristics as an indicator of chronic low-grade inflammation. A positive association between SIRI above 3.8 and the risk and poor prognosis of stroke (OR: 1.45, 95% CI: 1.10–1.91, *p *< 0.001; AUC: 0.626) was presented by Zhang *et al*. [121]. Interestingly, in Han *et al*. [122] study the higher SIRI values (above 1.02) were related with increased risk for future MACE (HR: 1.127, 95% CI: 1.034–1.229, *p* = 0.007; AUC: 0.624) in patients with acute coronary syndromes undergoing percutaneous coronary interventions and correlated with poor clinical presentation.

In cardiac surgery, postoperative values of SIRI measured on the 1st postoperative day following off-pump surgical revascularization above 5.4 (HR: 0.29, 95% CI: 0.09–0.92, *p* = 0.036; AUC: 0.616) were presented as a long-term mortality risk factor [98]. The preoperative values of SIRI >1.27 were postulated as 5 years mortality risk marker (HR: 6.16, 95% CI: 2.17–17.48, *p* = 0.012; AUC: 0.682) on group of 171 patients who underwent elective OPCAB revascularization [74].

## 12. Systemic Immune Inflammatory Index (SII)

Systemic index was based on NLR and thrombocytes as a possible marker of inflammatory and immunological activation simultaneously. It is calculated on total peripheral count of neutrophil and platelets divided by lymphocyte count. The significance for poor outcomes prediction after elective OPCAB revascularization was presented in Dey *et al*. [99] analysis. The increased incidence of atrial fibrillation, intra-aortic balloon pump requirement and ionotropic support was related to SII values ≥878 × ${\mathrm{10}}^{3}$/${\mathrm{mm}}^{3}$. In the latter results, the SII ≥878 ×${\mathrm{10}}^{3}$/${\mathrm{mm}}^{3}$, was found as independent poor outcomes predictor (OR: 1.010; 95% CI: 1.003–1.016, *p* = 0.003; AUC: 0.984) [99].

In diabetic patients referred for off-pump surgical revascularization, the postoperative values of SII >952 × ${\mathrm{10}}^{3}$/${\mathrm{mm}}^{3}$ (SII >952 (HR: 3.44, 95% CI: 1.02–11.66, *p* = 0.047; AUC: 0.698) were regarded as worse long-term prognostic factors [65]. Systemic index >545 × ${\mathrm{10}}^{3}$/${\mathrm{mm}}^{3}$ was proposed as postoperative atrial fibrillation risk factor (OR: 10.2; 95% CI: 5.1–20.2, *p *< 0.001; AUC: 0.91) in on pump surgical revascularization by Honue *et al*. [123]. Dogdus *et al*. [100] in their analysis presented SII value >935 × ${\mathrm{10}}^{3}$/mL as a better saphenous vein graft predictor than NLR (OR: 3.27, 95% CI: 1.94–5.65, *p *< 0.001).

The significant correlation between SII values (cutoff of 878 × ${\mathrm{10}}^{3}$/${\mathrm{mm}}^{3}$) and poor outcomes prediction following off-pump surgery was reported by Dey *et al*. [100] with 97.6% sensitivity, 91%, specificity, and AUC of 0.984. Presented analysis revealed also positive correlation between the SII values and length of mechanical ventilation and intensive care unit stay (R: 0.676; 0.527, *p *< 0.001).

## 13. Aggregate Index of Systemic Inflammation (AISI)

There is a scarce publication regarding aggregate index of systemic inflammation in coronary artery bypass grafting. The retrospective analysis performed on 538 patients undergoing elective off pump surgical revascularization did not reveal in multivariable analysis the relation between AISI preoperative and perioperative results and all -cause long term mortality [98].

AISI index above 221 (HR: 1.65; 95% CI: 1.36–2.01, *p* = 0.046; AUC 0.653) was reported as prolonged intensive care unit stay predictor in thoracic surgery [67] and above 798 (HR: 1.0001; 95% CI: 1.0000–1.0001, *p* = 0.029; AUC: 0.64) as mortality indicator in COVID-19 patients [124].

## 14. Limitations

First, the postoperative values of inflammatory markers presented in the review have to be separately applied for off-pump and on-pump patients due to differences between both groups related to surgical technique. Second, the simple inflammatory indexes obtained from peripheral blood count analysis are related to certain cellular components concentration changes. The derangements in neutrophil, monocyte, lymphocyte, and platelets counts are believed to be related to their activation. The activation can be measured directly by cytokines release or by membrane receptors activation. The simplified method presents the counts changes not activation by itself. Traditionally, we interpret count changes as cells’ activation, which is an appropriate logical relation in most of cases. Third, limitation is related to laboratory measurements as obtained results may be slightly different in different test related to normal range spread. Forth, the monocyte to lymphocyte ratio (MLR) and lymphocyte to monocyte ratio (LMR) are presented as interchangeable in different publications. Fifth, further studies are needed to establish role of inflammatory indices in particular groups of patients, especially diabetic, with kidney disease, with peripheral arterial disease.

## 15. Conclusions

Though OPCAB technique diminishes the risk for inflammatory activation, may still generate individual response that possess significant implication for long-term mortality. The surgery by itself can be regarded as a triggering factor for inflammatory response. Assessment of inflammatory indices obtained from the whole blood count analysis allows to indicate those patients who need scrupulous follow-up due to predicted worse long-term survival. Perioperative measurement and analysis of simple whole blood counts is inexpensive and easily available and may improve the results of surgical revascularization by better identification of patients at higher risk of worse outcomes.
